# Non-Vesicular Lipid Transport Machinery in *Leishmania donovani*: Functional Implications in Host-Parasite Interaction

**DOI:** 10.3390/ijms241310637

**Published:** 2023-06-26

**Authors:** Koushik Das, Tomoyoshi Nozaki

**Affiliations:** 1 Department of Allied Health Sciences, School of Health Sciences and Technology, University of Petroleum and Energy Studies, Dehradun 248007, India; 2 Department of Biomedical Chemistry, Graduate School of Medicine, The University of Tokyo, Tokyo 113-0033, Japan

**Keywords:** *Leishmania donovani*, lipid transfer protein, lipid signaling, drug development, pathogenesis

## Abstract

Eukaryotic cells have distinct membrane-enclosed organelles, each with a unique biochemical signature and specialized function. The unique identity of each organelle is greatly governed by the asymmetric distribution and regulated intracellular movement of two important biomolecules, lipids, and proteins. Non-vesicular lipid transport mediated by lipid-transfer proteins (LTPs) plays essential roles in intra-cellular lipid trafficking and cellular lipid homeostasis, while vesicular transport regulates protein trafficking. A comparative analysis of non-vesicular lipid transport machinery in protists could enhance our understanding of parasitism and basis of eukaryotic evolution. *Leishmania donovani*, the trypanosomatid parasite, greatly depends on receptor-ligand mediated signalling pathways for cellular differentiation, nutrient uptake, secretion of virulence factors, and pathogenesis. Lipids, despite being important signalling molecules, have intracellular transport mechanisms that are largely unexplored in *L. donovani*. We have identified a repertoire of sixteen (16) potential lipid transfer protein (LTP) homologs based on a domain-based search on TriTrypDB coupled with bioinformatics analyses, which signifies the presence of well-organized lipid transport machinery in this parasite. We emphasized here their evolutionary uniqueness and conservation and discussed their potential implications for parasite biology with regards to future therapeutic targets against visceral leishmaniasis.

## 1. Introduction

Eukaryotic cells are defined by the presence of a group of membrane-enclosed organelles or compartments having distinctive lipid and protein signature [1,2]. Each cell organelle performs a specific function by assuring proper segregation of the complex cellular processes catalyzed by an array of metabolic enzymes, structural and regulatory proteins [2]. Precise distribution of the proteins to different cellular compartments is ensured either by their intrinsic signal peptides or through post-translational modifications [2]. In contrast, such signal sequences are absent in lipid molecules to govern their intracellular translocation; however varying proportions of different lipid species are identified in different cell organelles [2,3,4]. Endoplasmic reticulum (ER) is the main site for lipid synthesis [5], from which lipids are then transported to their site of function. Studies have suggested that both vesicular and non-vesicular transport machinery come into play during the intracellular lipid trafficking and delivers the lipids to their target destinations [2]. Vesicular transport has a crucial role in protein trafficking, endocytic and exocytic (secretory) pathways [2]. It is an energy dependent process and involves cytoskeletal reorganization [2]. However, when vesicular transport was impaired either by depletion of ATP, reduced temperature, or treatment with specific pharmacological inhibitors (viz. colchicine and brefeldin A), lipid transport was still observed to be active among the organelles [6,7,8]. Moreover, Lipid transportation was detected among the cell organelles, not linked by vesicular transport mechanism (e.g., ER/mitochondria and ER/peroxisomes) [9,10]. These observations warrant that the non-vesicular transport also plays a significant role in intracellular lipid trafficking. Non-vesicular lipid transport between cellular compartments is greatly facilitated by LTPs, which ensure organelle-specific lipid distribution and cellular lipid homeostasis [2,4,11,12,13,14]. They can also locally modulate the lipid composition of membranes, thereby regulating a variety of cellular processes, viz. vesicular trafficking, lipid metabolism, and signal transduction [15,16,17,18,19]. Moreover, LTPs can also act as membrane contact sites (MCSs) between the endoplasmic reticulum (ER) and virtually all other cell organelles, and regulate the transport of Ca^2+^, metabolites, and lipids [2,17]. A typical eukaryotic cell possesses a repertoire of lipid molecules for three main functions, namely energy storage, cell compartmentalization, and cell signaling [20]. Each of the cell organelles possesses a variable concentration of different lipids, which provides a unique identity to each organelle and is required for optimum functions of organelles. For instance, ER has phosphatidylcholine (PC) (54%), phosphatidylethanolamine (PE) (20%), phosphatidylinositol (PtdIns) (11%), and cholesterol (Chol) (8%). The mitochondria have PC (37%), PE (31%), and cardiolipin (CL) (22%). In contrast, the plasma membrane (PM) contains Chol (34%) as one of its major lipids, along with sphingomyelin (SM) (17%) and phosphatidylserine (PS) (8%) [20]. Such organelle specific lipid compositions are determined by intracellular lipid transport and organelle specific in-situ lipid metabolism, and LTPs play an essential role in this process [2]. Chiapparino et al. has suggested that there are more than a hundred LTPs exist in humans and these can be divided into at least twelve protein families, including, fatty acid binding protein 2 (FABP2), Niemann-Pick disease, type C1 (NPC1) protein, Niemann-Pick disease, type C2 (NPC2) protein, Steroidogenic acute regulatory protein (StAR)-related lipid transfer (START) protein, Cholesteryl ester transfer protein (CETP), Oxysterol-binding protein (OSBP)-related proteins (ORPs), PtdIns transfer protein (PITP), Sterol carrier protein 2 (SCP2), Sec14 domain containing protein, Glycolipid Transfer Protein (GLTP), Synaptotagmin-like Mitochondrial-lipid-binding Protein (SMP) and repeating β-groove (RBG) domain containing protein [21,22,23,24].

**Figure 1:**
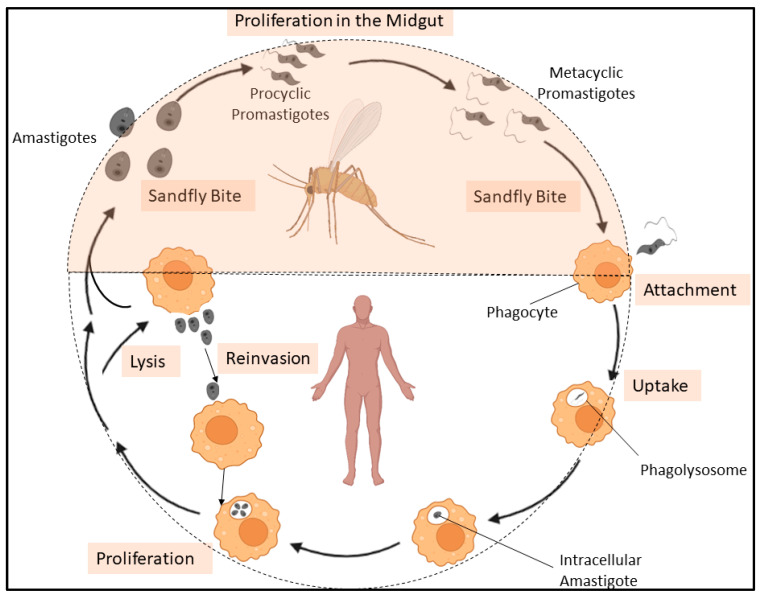
Schematic diagram illustrates the life cycle of *L. donovani*. *L. donovani* has two life cycle stages: the sandfly stage (**top**) and the mammalian stage (**bottom**). *L. donovani* proliferate in the midgut as procyclic promastigotes and differentiate into highly infectious metacyclic promastigotes. When a sandfly bites a mammalian host, the *L. donovani* promastigotes are injected into the dermis of the host. Then they are taken up by phagocytosis, once inside phagolysosomes of macrophages, they transform into amastigotes. Infected macrophages lyse and release amastigotes, which reinvade other macrophages to multiply further. The figure has been modified from Colineau, 2018 [30].

Visceral leishmaniasis (VL), caused by trypanosomatid parasite *Leishmania donovani*, infects annually 200,000–400,000 people annually and is prevalent in South Asian regions that includes India, Nepal, and Bangladesh, with reports of resurgence on the rise [25,26,27]. Such a resurgence of disease indicates the failure of current therapeutic intervention including, pentavalent antimonials, miltefosine, and paromomycin due to increased toxicity, limited efficacy and emergence of novel drug-resistant parasites. Liposomal amphotericin B has been used to treat VL. This lipid formulation is based on concept of targeted drug delivery to macrophages in the affected liver, spleen and bone marrow, which reduced the adverse effects including hypokalemia and nephrotoxicity. Three formulations including, liposomal amphotericin B (AmBisome^®^, Gilead Sciences, Inc., Foster City, CA, USA), amphotericin B lipid complex (ABLC; Abelcet^®^, Gilead Sciences, Inc., Foster City, CA, USA), and amphotericin B Chol dispersion (ABCD; Amphotec™, Gilead Sciences, Inc., Foster City, CA, USA) are available [28]. Moreover, there are no effective and safe vaccines available against the fatal parasitic disease and chemotherapeutic treatment of infected individual is one of the key strategies to control the disease [29]. Hence, development of a novel prophylactics and chemotherapeutics to control Leishmaniasis is required. However, lack of well-validated molecular targets in *L. donovani* has hindered the development of novel chemotherapeutics. *L. donovani* has two life cycle stages: the sandfly stage and the mammalian stage. The promastigote form of the parasite was found in sandflies and was injected into a mammalian host during a sandfly bite. Post-injection into a mammalian system, promastigotes were transformed into amastigotes form within the phagolysosomes of macrophages, which reinvade other macrophages to multiply further [30] (Figure 1). Identification of parasite-specific molecules, essential for intracellular growth, survival, and differentiation of *L. donovani* within host cells, could offer excellent intervention candidates for future therapeutics and also improve our understanding of host-parasite interaction. Lipids, an essential constituents of eukaryotic cell regulate a diverse array of cellular processes and have been implicated in cellular growth, differentiation, survival, adaptation under stress [31,32]. The essentiality of lipids and their effector molecules in parasite’s cell signaling, cell proliferation, host-parasite interaction and drug susceptibility have been reported in *Plasmodium falciparum* [33,34], *L. donovani* [35,36,37,38], *Trypanosoma cruzi* [39], *Entamoeba histolytica* [40,41] and *Giardia lamblia* [42,43]. However, molecular mechanisms of intracellular lipid trafficking and their implications in lipid homeostasis and in parasite’s biology have been greatly unexplored. Nevertheless, recent studies indicate that non-vesicular lipid transport by LTPs plays crucial roles in growth, survival and pathogenicity of protozoan parasites, *Plasmodium falciparum* [44,45] and *E. histolytica* [46,47], thus offers an excellent intervention candidate for future therapeutics. *Plasmodium* sp. induced extensive modifications to the infected host cell, which included the formation of a membranous structure (parasitophorous vacuole, or PV) around the intracellular parasite. Therefore, to maintain a finely tuned and dynamic lipid environment, the organisation and distribution of lipids to different cell sites requires specialised LTPs [48]. Previous studies have identified a phospholipid transfer protein in *P. falciparum* (PFA0210c) that is present in the PV during growth and is later recruited to organelles in the parasite [44,45]. Furthermore, a MCS has been identified between the parasitophorous vacuolar membrane (PVM) and the parasite plasma membrane (PPM) within the PV. This contact site facilitates the transport of proteins, lipids, nutrients, and metabolites between the cytoplasm of the parasite and the cytoplasm of the host erythrocyte (RBC) and a lipid transporter of *P. falciparum* (PfNCR1) is localised at the contact site and participates in the transport process [49]. In contrast, LTP plays different roles in another protozoan parasite, *E. histolytica*. The identified phospholipid transfer proteins mostly regulate receptor-ligand mediated signaling processes during host-parasite interaction, such as trogocytosis and/or phagocytosis of host cells, pinocytosis of fluid-phase nutrients, and exocytosis and/or secretion of virulence factor, cysteine proteases (CPs) [46,47]. In the present study, we have a repertoire of LTP homologs in *L. donovani* based on a domain-based search on TriTrypDB coupled with bioinformatics analyses, which suggests the presence of well-organized lipid transport machinery in this parasite. We outlined their evolutionary uniqueness and conservation, and discussed their potential implications for leishmanial biology in regards to future therapeutic targets against VL.

## 2. Overview on Domain Architecture of Lipid Transfer Proteins (LTPs)

LTPs markedly enhance the magnitude of non-vesicular lipid transport between the donor and acceptor membranes. Their lipid transport was facilitated by special lipid-transfer domains (LTDs) which can bind and accommodate a variety of lipid ligands inside their hydrophobic binding cleft [17]. For instances, the lipocalin domain of the FABP2 protein (interacts with palmitate); the NTD of the NPC1 protein (interacts with Chol); the ML domain of NPC2 protein (interacts with Chol sulfate); the START domain of START protein (binds to either sterols, phospholipids, or ceramides); the LBP/BPI/CETP domain of the CETP protein (interacts with two molecules each of cholesteryl ester and PC); oxysterol-binding protein (OSBP)-related domain (ORD) of ORP (binds to sterols and PtdIns4P); PITP domain of PITP protein (binds to PC, PtdIns and PIs); SCP2 domain of SCP2 protein (interacts with palmitate); Sec14 domain of Sec14 protein (binds to PC and PtdIns); GLTP domain of GLTP protein (complex with lactosylceramide) [21,22]. The SMP domain is a newly identified lipid transfer protein that functions at MCSs between ER and plasma membrane (PM) and facilitates the lipid transfer by interacting via its tip region with extremely curved subdomain of tubular ER and the acidic-lipid enriched PM. Disruption of these mechanisms results in a defect in autophagosome biogenesis contributed by extended synaptotagmin (E-Syt) [22]. Recently, a new type of bridge-like lipid transfer domain with long hydrophobic grooves has emerged. These long hydrophobic grooves are made up of multiple repeating modules, consisting of five β-sheets followed by a loop and hence, named RBG domain. The RBG domain has been identified in VPS13, ATG2, SHIP164, Csf1, and the Hob proteins [23,24]. Binding of specific lipid molecules is influenced by several factors viz. hydrogen bonds and hydrophobic interactions, that help nestle the molecules in the pocket [2]. Additionally, LTPs often contain a range of “organelle targeting” domains/motifs such as protein kinase C (PKC) conserved 1 (C1), PKC conserved 2 (C2), FFAT motifs, GOLD domain, Ankyrin repeats, RhoGAP (Rho GTPase activating protein) domain, Phox homology (PX) domain, FYVE (Fab-1, YGL023, Vps27, and EEA1) domain and pleckstrin homology (PH) domains for guiding them to specific cellular compartments [17]. The C1 and C2 domains have been identified on various proteins, including protein kinase C (PKC), phospholipase A2 (PLA2), phospholipase C (PLC), phospholipase D (PLD), and phosphoinositide (PtdIns phosphate, PI) 3-kinase, with varying specificities towards phospholipids [50]. However, their organelle targeting was Ca^2+^ ion-dependent [50]. FFAT motifs bind with VAMP-associated protein (VAP) protein family of ER. GOLD (for Golgi dynamics) domain regulates the Golgi function and secretion. Ankyrin repeats are critical for protein folding and stability. RhoGAP domain catalyzes the hydrolysis of GTP that is bound to Rho, Rac and/or Cdc42, thus inactivating these regulators of the actin cytoskeleton, PX domains have emerged as membrane interacting domains, bind to PtdIns and phosphoinositides (PtdIns phosphate) and FYVE are the membrane-targeting domains, highly specific for PtdIns-3-phosphate (PtdIns(3)P) [17]. The LTPs can facilitate intra-cellular transport of lipids through a sequential process involving interaction of the LTPs with the donor membrane [16] followed by the opening of the lipid binding cavity, extraction of lipids from the donor membrane, dissociation of the LTPs from the donor membrane in a “closed” conformation, and transport of the LTPs through the cytoplasm towards the acceptor membrane. The transport is completed by the interaction of LTP with the acceptor membrane, followed by the opening of the lipid binding cavity, and the desorption of lipid molecules [16,51,52,53,54,55,56,57]. LTPs with “organelle targeting” domains/motifs for two different organelle membranes can perform efficient lipid transport at MCSs between these two cellular compartments [2,9,17,58,59,60,61].

## 3. Comparative Analysis of LTP Subtypes between *L. donovani* and Other Organisms

### 3.1. Identification, Domain Organization of LTP Homologs in L. donovani BPK282A1

**Figure 2:**
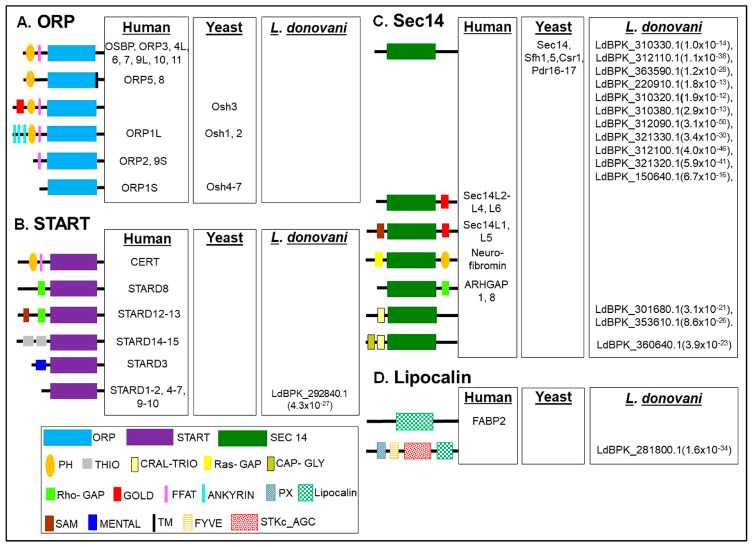
Classification and domain organization of lipid transfer proteins (LTPs) from human, yeast, and *L. donovani* BPK282A1. Based on domain organization and cellular localization, LTPs are grouped into cytosolic and membrane bound LTPs. Cytosolic LTPs have only lipid transfer domains (LTDs) such as, (**A**) oxysterol-binding protein (OSBP)-related domain (ORD, binds to sterols and to PtdIns4P), (**B**) steroidogenic acute regulatory protein (StAR)-related lipid transfer (START) domain (binds to either sterols, phospholipids, or ceramides), (**C**) Sec14 domain (bind to PC and PtdIns), and (**D**) Lipocalin domain (binds to palmitate), all of which can accommodate the hydrophobic moieties of various lipid ligands from aqueous environment of cytoplasm. Membrane bound LTPs possess various combinations of LTDs with other additional membrane-anchored domains/motifs such as pleckstrin-homology (PH) domain, diphenylalanine-in-an-acidic-tract (FFAT) motif, Golgi dynamics (GOLD) domain, Ankyrin repeats, Phox homology (PX) domain, FYVE (Fab-1, YGL023, Vps27, and EEA1) domain etc. and function as membrane contact sites (MCSs). *L. donovani* has sixteen (16) potential LTP homologs including one START candidate, fourteen Sec14 candidates and one Lipocalin domain containing protein. *L. donovani* lacks homolog of eukaryotic OSBP-related proteins (ORPs) and other eukaryotic proteins, discussed in Section 3.1.3. TriTrypDB ID of *L. donovani* BPK282A1 LTP homologs are shown. The e-values for each of the identified LTP homologs in *L. donovani* BPK282A1 genome are shown after the TriTrypDB ID of each LTP homolog.

**Figure 3:**
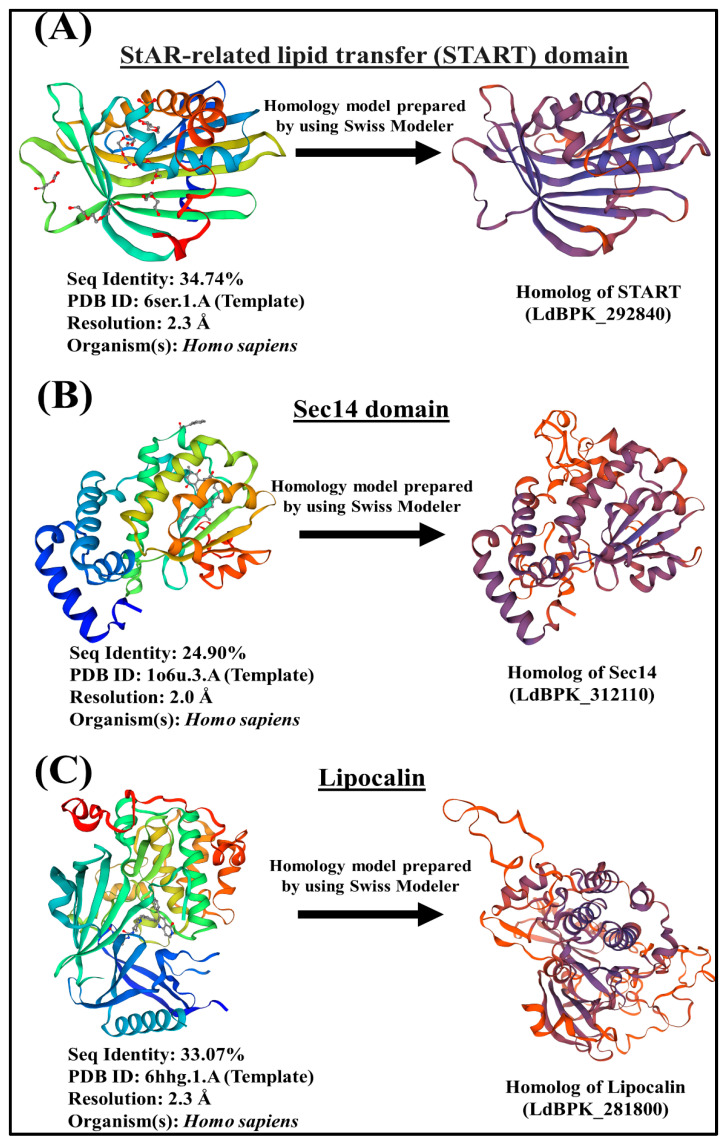
Modeling of the 3D structure of representative LTP candidates of *L. donovani*. The protein sequences of lipid transfer domains (LTDs) of representative homologs from each of the classified groups [i.e., (**A**) START, (**B**) Sec14 and (**C**) Lipocalin] were used for protein homology modelling. The PDB ID of each template and TriTrypDB ID of representative homologs from amastigote form of *L. donovani* are shown. The structural resolution of the Protein Data Bank structures (2.3 Å for PDB ID: 6ser.1.A, 2.0 Å for PDB ID: 1o6u.3.A and 2.3 Å for PDB ID: 6hhg.1.A) have been provided. The representative homologs (i.e., LdBPK_292840, LdBPK_312110 and LdBPK_281800) from each classified groups shared weak homologies with their counterparts from higher eukaryotes, indicating their potentiality as parasite-specific intervention candidates.

InterPro domain search (PFAM) analysis on TriTrypDB version 53 (released 21st July 2021) has enabled us to identify the potential LTP candidates in *L. donovani* BPK282A1. The *L. donovani* BPK282A1 genome contains assorted repertoire of sixteen (16) potential lipid transfer protein (LTP) homologs [one steroidogenic acute regulatory protein-related lipid transfer (START) proteins, fourteen Sec14 like proteins and one Lipocalin domain containing protein] (Figure 2) with different E-value (as E-value provided by TriTrypDB). The STARD domain of START protein, Sec14 domain of Sec14 protein and lipocalin domain of FABP2 protein are used for homology analysis, which identified the candidate genes shown in Figure 2. The e-values for each of the identified LTP homologs in *L. donovani* BPK282A1 genome are shown in Figure 2. Further verification for the presence of each of the indicated lipid transfer domain (LTD) [for instances, STARD, Sec14 and Lipocalin] in these sixteen potential LTP homologs was done by NCBI conserved domain search analysis and position of individual domain in each homolog was defined. Next, these sixteen potential LTP homologs were compared with LTP homologs previously reported in other eukaryotes like human, *Saccharomyces* sp. and were then grouped based on their domain organization (Figure 2). Information on non-vesicular lipid transport is mostly available in human [62], yeast [63,64,65], plant [66], in *Plasmodium* sp. [53,54] and *Entamoeba histolytica* [39,40]. The modelling of the 3D structure of representative LTP candidates of *L. donovani* (LdBPK_292840, LdBPK_312110 and LdBPK_281800) from each of the identified group (i.e., START, Sec14 and Lipocalin respectively) were analyzed, which reveals that studied LTP homologs from each classified groups shared weak homologies with their counterparts from human, indicating their possibilities as parasite-specific intervention candidates (Figure 3).

#### 3.1.1. START Domain Containing Proteins

*L. donovani* possesses only one steroidogenic acute regulatory protein-related lipid transfer (START) domain containing protein (LdBPK_292840.1). Apart from its lipid sensing domain, this sterol specific LTP lacks any potential membrane anchoring domain indicating their cytosolic localization (Figure 2). *L. donovani* START protein homolog possess a deep lipid-binding pocket (Figure 3), which can accommodate lipid moiety and transfer between the intracellular compartments. It could modulate the local lipid metabolism on certain organelle membrane by delivering lipids to organelle residing lipid biosynthesis enzymes and also could participate in signaling cascades [2,67]. Previous study suggests that dynamic changes in sterol composition influences the virulence and stage conversion of *Leishmania* spp. [68]. Furthermore, Sterol methyltransferase (SMT), involved in the synthesis of parasite-specific C24-methylated sterols, including ergosterol and 5-dehydroepisterol is crucial for optimal mitochondrial function and virulence in *L. major* [69]. Since, LTPs are an integral part of lipid metabolic machinery [2], it could be conceivable that the identified START homolog (LdBPK_292840.1) could contribute in the process of sterol metabolism and dynamics in *L. donovani*.

#### 3.1.2. Sec14s

*L. donovani* genome contains fourteen (14) potential Sec14 homologs (Figure 2). Eleven (11) of which possess only Sec14 domain, similar as their counterparts in yeast (Figure 2). Another three homologs have additional functional domains along with Sec14 domain, unique to *L. donovani.* Two candidates (LdBPK_301680.1 and LdBPK_353610.1) contain additional CRAL-TRIO domain, while one candidate (LdBPK_360640.1) possesses both CRAL-TRIO domain and Cytoskeleton-associated protein-glycine-rich (CAP-Gly) domain along with Sec14 domain (Figure 2). The Sec14 domain binds the hydrophobic tail of a single lipid molecule, positioned in the middle of the protein and carry out intracellular lipid transport. First identified in budding yeast, Sec14 is reported to be essential for secretory protein transport from the Golgi complex to other biological membranes [46,70,71,72]. In humans, twenty-nine Sec14 homologs have been identified most of which possess additional Ras-GAP or Rho-GAP domain (Figure 2) implying that small G protein regulation may be a functional theme of many Sec14 proteins [62,70,71,72]. The CAP-Gly domains are the protein-interaction modules, bind to C-terminal EEY/F-COO^−^ sequence motifs of α-tubulin and other microtubule-associated protein, other structural elements including end-binding homology domains, zinc-finger motifs and proline-rich sequences and involved in the maintaining of cell architecture and signaling [21,22].

#### 3.1.3. Lipocalin

*L. donovani* genome contains single homolog (LdBPK_281800.1) of Lipocalin (Figure 2). It possesses three additional membrane binding and/or catalytic domains namely, PX domain (binds to PtdIns and/or PtdIns phosphates or phosphoinositides of endocytic system) [17], FYVE domain (binds specifically to PtdIns(3)P of endosomal membrane) [17] and catalytic domain of AGC family Serine/Threonine Kinases (STKc_AGC) along with the lipocalin domain (Figure 2). The Serine/Threonine Kinases (STKs) catalyze the transfer of the gamma-phosphoryl group from ATP to serine/threonine residues on protein substrates. AGC kinases regulate many cellular processes including division, growth, survival, metabolism, motility, and differentiation. Such domain organization of LdBPK_281800.1 is unique to *L. donovani*, which might enable the candidate to regulate the endosomal trafficking and related signaling processes via interacting with phosphoinositides and other lipid effectors on endosomal membrane [17].

#### 3.1.4. Other Proteins Known to Be Involved in Lipid Transfer in Other Organisms, but Missing in *L. donovani*

*L. donovani* lacks a significant number of LTP homologs, known to be present and functional in other eukaryotes (Figure 2). *L. donovani* has no homologs for human PITPs, similar as in *Saccharomyces* sp. [73,74] and in parasitic protist, *E. histolytica* [46]. *L. donovani* possesses a START domain protein homolog, which could potentially function as PITP, as reported in *Saccharomyces* sp. [64], *Plasmodium* sp. [44,45] and *E. histolytica* [47]. *L. donovani* lacks a homolog of eukaryotic ORPs. In yeast and humans, cytosolic ORPs mediate sterol transport between the ER and other organelles whereas the ORPs with additional PH and FFAT domains could facilitate ER-Golgi lipid transport by interacting with ER-resident proteins, vesicle-associated membrane protein-associated proteins (VAPs) in humans and with Scs2p in yeast [75]. ORPs also interacts with Rab GTPases and controls the intra-cellular movement of transport vesicles, as reported for ORP1L in higher eukaryotes [2,76,77]. Since the *L. donovani* genome encodes none of ORP homologs (Figure 2), it is plausible that few of the identified Sec14 candidates could regulate Golgi mediated vesicular trafficking and exocytosis on their behalf by maintaining the critical balance in DAG and PC levels in Golgi complex, as reported in *S. cerevisiae* [2,64]. The genome of *L. donovani* also lacks the homologs of protein of relevant evolutionary and lymphoid interest (PRELI). PRELI proteins are involved in the transportation of phospholipids such as, CL and PE between the inner (i.e., inner mitochondrial membrane, IMM) and outer mitochondrial membrane (i.e., outer mitochondrial membrane, OMM) and regulate mitochondrial lipid homeostasis [78,79]. They interact with membrane organizing proteins, participate in MCSs and maintain the structural organization of mitochondria [78,79]. Mitochondrial PRELI protein of *Toxoplasma gondii* (TgPRELID) is associated with multidrug resistance of the parasite [80]. However, the study also suggested that some START domain containing proteins with mitochondrial targeting signals have functional redundancy and be could involve in mitochondrial lipid transport and homeostasis [81]. Since, *L. donovani* lacks homologs of PRELI proteins but possesses homolog of START domain protein, it might be implicated in intramitochondrial lipid homeostasis. *L. donovani* also lacks homologs of eukaryotic synaptotagmin-like, mitochondrial, and PH domain (SMP) containing proteins [82], localized at the ER–mitochondria encounter structure (ERMES) and at other MCS in yeast [17]. *L. donovani* lacks the homologs of the ML domain of the bovine NPC2 (binds with Chol sulfate), the LBP/BPI/CETP domain of the CETP (interacts with two molecules each of cholesteryl ester and PC), the SCP2 domain of the yellow fever mosquito SCP2-like 3 (binds with palmitate), the NPC1 NTD of the NPC1 (interacts with Chol) and the GLTP domain of the GLTP (binds lactosylceramide) [21,22].

### 3.2. mRNA Expression of LTP Homologs L. donovani BPK282A1

**Figure 4:**
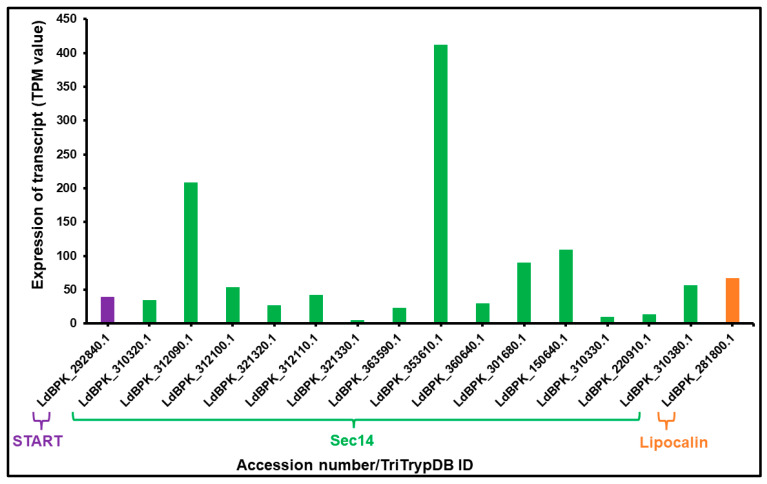
Relative mRNA expression of LTP homologs in *L. donovani* BPK282A1 amastigotes. Survey of *L. donovani* BPK282A1 genome has a repertoire of sixteen (16) LTP homologs. The levels of mRNA expression are shown with TPM (transcript per million) value as per TriTrypDB. The levels of mRNA expression of LTP candidates, shown in the figure are obtained from amastigotes form of *L. donovani* parasite. Two members of *L. donovani* Sec14 homologs (LdBPK_353610.1 followed by LdBPK_312090.1) show the higher mRNA expression in BPK282A1 strain among all identified LTP candidates, while another two Sec14 homologs (LdBPK_321330.1 and LdBPK_310330.1) exhibits the minimal mRNA expression in BPK282A1 strain among all candidates. The identified START (LdBPK_292840.1) and lipocalin (LdBPK_281800.1) homolog show moderate level of mRNA expression.

Relative steady-state levels of mRNA expression of a panel of sixteen (16) potential LTP homologs from *L. donovani* (one START protein, fourteen Sec14 domain proteins and one lipocalin domain containing protein) were investigated using data (TPM value) available at TriTrypDB. Two members of Sec14 protein homologs showed higher mRNA expression in BPK282A1 compared to other LTP candidates (in a descending order of LdBPK_353610.1 and LdBPK_312090.1) (Figure 4). On the other hand, LdBPK_321330.1 and LdBPK_310330.1, these two Sec14 homologs show very low levels of expression (Figure 4). This may indicate their potential roles in growth and stage differentiation process in *L. donovani*, as previously shown for LTPs in *E. histolytica* [47,83] and during somatic embryogenesis and stress management in *Arabidopsis thaliana* [84]. The START (LdBPK_292840.1) and lipocalin (LdBPK_281800.1) homolog of *L. donovani* showed moderate level of expression compared to other identified LTP candidates (Figure 4).

## 4. LTPs and Their Biological Implications

### 4.1. Previous Reports on Biological Importance of LTPs in Other Eukaryotic Systems

The biological roles of the LTPs are diverse. LTPs execute vectorial (often bidirectional) transport of lipids between cell organelles [17]. For instance, steroidogenic acute regulatory protein-related lipid transfer (START) domain containing proteins with varying lipid specificities can facilitate inter-organelle lipid transport. The STARD1 sub-family (containing STARD1 and STARD3/MLN64) and STARD4 sub-family (comprised of STARD4, STARD5, and STARD6) help in Chol transport to various organelles [67]. The STARD2 sub-family (containing STARD2, STARD7, STARD10, and STARD11/CERT) regulates either PC/PE or CERT from ER to the Golgi complex [67]. LTPs can function as lipid presenting proteins for lipid metabolizing enzymes [46,85,86,87] and participate in signal transduction [87,88,89,90]. For instance, PtdIns transfer proteins (PITPs) can transport PtdIns, the main precursor of PIs, and their metabolizing enzymes (such as PI 4-kinases, PtdIns4P 5-kinases, PI 3-phosphatases, PI 5-phosphatases, and non-specific phosphatases) from the ER to the cell organelles. Phosphatidic Acid (PA), produced from diacylglycerol (DAG), was transported back to the ER from the plasma membrane (PM) for replenishment of the PtdIns pool [87,91,92,93,94]. LTPs, post-interaction with lipid molecules, can function as lipid sensors, exhibiting altered affinity to other associated proteins in bio-membranes [2]. For example, Sec14 homolog can sense an increased level of PC in Golgi, regulate PC production from DAG through the cytidine diphosphate (CDP)-choline pathway, and thus maintain a critical balance in DAG and PC levels in Golgi, which is crucial for Golgi-mediated vesicular trafficking and exocytosis in *S. cerevisiae* [2,64,95,96,97,98,99,100]. Oxysterol-binding-protein-related proteins (ORPs) control the intracellular movement of transport vesicles by interacting with Rab GTPases [2,74,76]. PITP mediates a transient alteration in lipid (e.g., PtdIns (4,5) P2) distribution at the site of exocytosis, which is required for priming and docking of the exocytic vesicle complex with the PM [2,92]. LTPs can regulate the nuclear transport of lipid precursors and nuclear associated functions [31,101,102,103,104,105,106,107,108,109,110,111,112,113,114]. For instance, microsomal triglyceride transfer protein, which is involved in lipid homeostasis, can interact with hepatocyte nuclear factor 4 (HNF4), RNA helicase DDX3 and small heterodimer partner (SHP) [115]. ORP8, the sterol transporter can interact with a nucleopore component (Nup62) and regulate the nuclear transport [116,117]. Moreover, ORP3 and ORP7 interact with R-Ras and regulate cytoskeleton organization, cell adhesion, and migration [118,119]. STARD8/12/13 (START proteins with Rho-GAP domain) regulates the cytoskeleton organization and migration of a cancer cell line [67].

### 4.2. Biological Significance of LTPs in L. donovani

#### 4.2.1. Previous Reports on Lipids Homeostasis and Their Implications in *Leishmania* sp.

Compared to other protozoan parasites for instances, in Entamoeba histolytica [41], Toxoplasma gondii [80] and Plasmodium falciparum [33,34], available reports on biological significance of lipid signaling and metabolism in L. donovani are limited. Phospholipids (PL) have been reported as the predominant class of lipid in *Leishmania* sp. The fatty acid (FA) composition was characterized by a lower abundance of the precursor C18:2(n-6) [36,120]. *Leishmania* sp. utilize lipids and FA as major energy source during stage-specific conversion [121,122]. *Leishmania* sp. can either synthesize lipids and FA de-novo or scavenge from the host. Promastigote, the extracellular form of the parasite relies on de-novo synthesis of lipids (specifically, glycerophospholipid, sterols and sphingolipids), while intracellular amastigotes form largely depends on host-derived lipids for intracellular growth and survival [35]. Lipid droplets (LDs), which are observed both inside the parasitophorous vacuole and within parasite cytoplasm may indicates that *L. donovani* could use LDs as high energy sources for their growth [36]. The functional implications of exosomes from *L. donovani* during host-parasite interaction was also reported [123,124,125,126,127]. The synthesis, intracellular transportation, and secretion of exosomes by parasite bodies also depends on lipid signaling and turn over, as reported in *Giardia lamblia* [128] and higher eukaryotes [129,130]. Moreover, the entry of promastigotes into the host cells, after flagellar regression, requires extracellular lipases to disrupt the host membrane. Inside host cell, secreted lipases from the amastigote would turnover lipid, which then gains channel-based entry to fuel amastigote metabolism [131,132,133]. A recent preliminary work has identified a novel lipase in both *L. donovani* and *L. major*, LdLip3 [37]. It has been shown that lipase precursor like protein promotes miltefosin tolerance in *L. donovani* by increasing parasite infectivity [38]. Host lipid profiles are also reported to be in close association with infection. Hyperlipidemia protects against *L. donovani* infections through membrane Chol [134]. On the other hand, mice and patients infected with leishmanial parasites exhibit decreased level of serum Chol [135,136]. The impairment of host phosphatidyl-linositol 3-Kinase (PI3K) signaling ensures parasite’s intracellular persistence [137]. In contrast, PI3Kγ knockout mice showed significantly enhanced resistance against *L. mexicana* [138].

#### 4.2.2. Proposed Implications of LTPs in the Biology of *L. donovani*

LTPs could participate in the diverse array of biological processes in metazoan, as discussed earlier. However, in this section, we have discussed their proposed implications in biology of both promastigotes (i.e., extracellular form in sandfly) and amastigotes (i.e., intracellular form in vertebrates host) forms of *L. donovani*.

##### Role of LTPs in the Regulation of Vesicular Trafficking and Secretion of Virulence Factors

Exoproteome analysis of *L. donovani* revealed that the parasite secretes a repertoire of secretory molecules including, molecules for immunomodulation and evasion [glycoprotein 63 (gp63), elongation factor 1α (EF1α), oligopeptidase, proteophosphoglycan (PPG)], increased pathogenicity [chitinase, casein kinase, KMP-11, cysteine peptidase and metalloproteases], and parasite protection [tryparedoxin peroxidase (cTXNPx)], which influence the pathophysiology of the disease by induction of inflammatory cytokines, particularly IL-17a [139,140,141,142,143,144,145,146,147,148,149,150,151,152,153,154,155] and/or implicated in drug-resistance in parasites [155]. Exosomes secreted by drug-resistant parasite form a nucleosome complex with human histone in host chromatin during the progression of the disease [156]. Nevertheless, LTPs can function as lipid sensors to regulate Golgi-mediated (or post-Golgi) vesicular trafficking, as described for Sec14 in *Saccharomyces cerevisiae* [18,70,156,157]. The identified Sec14 homologs, which possess only Sec14 domain (eleven among sixteen identified LTP homologs) could sense the lipid composition of Golgi membrane and regulates ER-Golgi lipid transport, thus could control the trafficking of the transport vesicles and other Golgi associated functions, as observed in *S. cerevisiae* [18,70,156,157]. Moreover, the priming and docking of secretory vesicles with the exo-cyst complex that tethers at the site of exocytosis on the plasma membrane (PM) require reorganization of PIs, specifically PI(4,5)P2 at target sites [158]. The identified START homolog could trigger such in-situ reorganization of PIs on PM by extracting and delivering PIs at target sites, which in-turn promotes the exocytosis process, as suggested in other parasites including, *E. histolytica* and *Trichomonas vaginalis* [47,159]. Moreover, the identified lipocalin homolog of *L. donovani* (LdBPK_281800.1) with endosomal membrane phosphoinositides targeting modules (i.e., PX, FYVE) could also regulate the endosomal trafficking and signaling cascades [17].

##### Implications of LTPs in Receptor-Ligand Interactions (Nutrient Sensing), Cytoskeleton Reorganization and Motility of the Parasite

*Leishmania* parasite largely depends on their ability to sense and respond to ever-changing host derived micronutrients for successful navigation through its life cycle. However, *Leishmania* parasite largely depends on flagellar membrane proteins and downstream signal transduction system like, glucose/hexose transporter 1 (GT1) and TOR3 signaling pathway [160,161], aquaglyceroporin 1 (AQP1) and MAPK2 pathway [162], Arginine permease 3 (AAP3) and MAPK2 [163] for sensing of host micronutrient and associated transcriptional regulation. Adenylate cyclase (AC) and cAMP mediated signaling on flagellar membrane also stimulates the innate immune response of host [164]. Above receptor-ligand mediated signaling processes, followed by asymmetric distribution of PLs at plasma membrane require substantial transport and turn-over of lipids and LTPs (for instance, identified START homolog) could plays important role, as suggested in other protozoan parasites, for instance, *E. histolytica* [47], *Plasmodium* sp. [44,45] and other eukaryotic system [165]. Modulation of in-situ lipid metabolism is also important for actin-mediated cytoskeleton reorganization and flagella-dependent motility of promastigotes [166,167,168,169,170]. START domain containing LTP (LdBPK_292840.1) could regulate the cytoskeleton organization and motility of parasite, as START homologs (STARD8/12/13) are involved in cytoskeleton organization and migration of cancer cell line [89,171,172]. The identified Sec14 homolog of *L. donovani* with CAP-Gly domain (LdBPK_360640.1) could regulates the cytoskeleton reorganization and motility of the parasite, as CAP-Gly domain interacts with the C-terminal EEY/F-COO^−^ sequence motifs of α-tubulin and other microtubule-associated protein [21,22].

##### Implications of LTPs in Intracellular Survival of *L. donovani* within Macrophages

Post-internalization of promastigotes by macrophages, intramacrophagic transformation of promastigotes into amastigotes requires substantial changes in lipid and FA compositions [120]. The free FAs, Chol and PLs (SM and PS) were increased, while triglycerides, ergosterol and PLs (PtdIns and lysoPE) were decreased during transition [68,120]. The identified Chols are obtained and transported from the host (i.e., host-derived lipid) as *Leishmania* spp. lack the enzymes for Chol synthesis [68], while identified ergosterol may be synthesized by sterol methyltransferase (SMT) of *Leishmania* sp. [69]. Such stage-specific conversion of lipid composition also requires in-situ synthesis and turn-over of various lipid classes and LTPs could facilitates the local lipid metabolism in given organelle by extracting and delivering of lipid moieties to lipid metabolic enzymes [2]. *L. donovani* substantially depends on the autophagy protein Atg8 for infection and survival under stress [109,123,124,125,126]. LTPs could potentially regulate the functional efficiency of Atg8 by facilitating the recruitment of lipid effectors, as observed in protozoan parasite *Entamoeba* sp. [36,128,129,130]. *Leishmania* promastigotes can either inhibit phagosome maturation by accumulating F-actin in the periphagosome [173], retaining actin polymerization machinery components such as Arp2/3, Wiskott-Aldrich Syndrome Protein (WASP), -actinin, Myosin II, and Nck in the phagosome [174] or prevent the phagolysosome biogenesis by improper recruitment of signaling effectors, including Rab7, LAMP-1, followed by poor interaction with late endosomes and lysosomes [29]. Time-dependent recruitment of these downstream effectors during phagosomal maturation requires transient synthesis and site-specific enrichment of PLs on a given bio-membrane and LTPs, being a part of the lipid biosynthesis machinery, could facilitate the local lipid metabolism, as described earlier [2]. LTPs could support the formation of parasitophorous vacuole, transfer of PL to the membrane of parasitophorous vacuole, and provide a safe niche for successful growth and differentiation of promastigotes within the host cells, as reported in *P. falciparum* [45]. Furthermore, LTPs could support the function of *L. donovani* secretory lipases to provide lipid precursors for amastigote metabolism [37], as suggested earlier [2]. The abundance of PL and Triacylglycerols (TAG) was also shown to regulate parasite’s sensitivity towards miltefosine drug in *L. major* [29].

## 5. Concluding Remarks

We have identified a repertoire of LTPs in *L. donovani* by a domain-based survey of LTP homologs in TriTrypDB. The genome of *L. donovani* possesses single homolog of START domain protein, which could potentially be implicated in inter-organellar lipid transport from ER to various cell organelles [67,89,90] and/or could regulate the cytoskeleton organization, and motility, as described in other higher eukaryotes [67]. The presence of single homolog of START domain protein in *L. donovani* might indicates its potential ability to perform diverse biological functions, usually regulated by different START protein candidates in other eukaryotes [67,89,90]. The genome of *L. donovani* also has multiple homologs (fourteen) of Sec14 domain containing protein. Eleven (11) of them with solely Sec14 domain, similar in yeast could function as lipid sensors [96,97,98] and maintain the critical level of DAG and PC in Golgi, thus potentially regulate the Golgi-mediated vesicular trafficking and secretion of important secretory molecules, including cystein- or metalloproteases, nuclease, PPG, chitinase, gp63, cTXNPx etc., implicated at various stages of Leishmaniasis, as described earlier [2,63,64,70,74,76,98,99,100]. Sec14 homolog with CAP-Gly domain could potentially regulates the cytoskeleton reorganization and associated signaling, as described earlier [21,22]. The lipocalin homolog with endosomal membrane phosphoinositide targeting domains (i.e., PX, FYVE) could regulate the endosomal trafficking [17]. However, the genome of *L. donovani* lacks the important homologs of PITPs, which are known to regulate the in-situ lipid metabolism in a cell by providing precursors to metabolic enzymes and also participate in receptor-ligand interaction and the signal transduction process during host-parasite interaction at the cell periphery [87]. The identified START in *L. donovani* could potentially function as PITP, as reported in *Saccharomyces* sp. [175], *Plasmodium* sp. [44,45] and *E. histolytica* [47]. *L. donovani* lacks the homologs of ORPs, regulates the intracellular movement of transport vesicles and exocytosis [2,78,79]. The identified Sec14 homologs could have functional redundancy as described in *Saccharomyces* sp. [2,96]. *L. donovani* genome do not possesses homologs of PRELI protein, which regulates the mitochondrial lipid transport in higher eukaryotes [78,79]. However, it could be interesting to explore mitochondrial lipid transport machinery in parasitic protists, as they could be significantly different from higher eukaryotic systems due to their earlier evolutionary position. Furthermore, any potential LTP homologs with nuclear localization sequence (NLS) and nuclear export sequence (NES) have not been identified in the genome of *L. donovani* in compare to other parasitic protists [39]. Such LTPs with potential NLS and NES have the ability of nucleocytoplasmic translocation and could be involved in nuclear lipid transport and lipid homeostasis. The genome of *L. donovani* lacks homologs of SMP domain containing proteins, which are organized at various MCSs and ERMES complexes in other eukaryotic systems [17]. *L. donovani* genome also lacks the homologs of the ML domain of the bovine NPC2 (binds with Chol sulfate), the LBP/BPI/CETP domain of the CETP (interacts with two molecules each of cholesteryl ester and PC), the SCP2 domain of the yellow fever mosquito SCP2-like 3 (binds with palmitate), the NPC1 NTD of the NPC1 (interacts with Chol) and the GLTP domain of the GLTP (binds lactosylceramide) [21,22]. Detailed molecular level studies are required to elucidate the functional implications of individual LTPs in the biology and pathogenesis of this parasite. Once parasitic-specific LTPs and their essentiality in the biology and virulence of parasites will be identified, they could provide novel therapeutic targets against this medically relevant pathogen. The majority of the available chemotherapeutics against lipid signaling cascades target lipid biosynthetic enzymes [176,177], such molecules often show specific but limited effects on a eukaryotic system such as in malignant cells. The parasitic organisms have excellent bypass mechanisms to overcome such inhibitory effects of drug molecules, resulting in the generation of drug-resistant pathogens. In contrast, chemical inhibition of parasite-specific LTPs could be effective against drug-resistant parasites, because specific LTP molecules regulate a wide range of cellular processes which can impact parasite biology greatly, as described above.

## Data Availability

All the data are available on request.

## Abbreviations

acyl-PG	acyl-phosphorylglycerol
BA	β-amylase
CL	cardiolipin, 1,3-bis(*sn*-3′-phosphatidyl)-*sn*-glycerol
DAG	diacyl glycerol
ER	endoplasmic reticulum
ERMES	ER–mitochondria encounter structure
HNF4	hepatocyte nuclear factor 4
IMM	inner mitochondrial membrane
LTD	lipid transfer domain
LTP	lipid transport protein
MCS	membrane contact sites
OMM	outer mitochondrial membrane
ORP	oxysterol-binding-protein-related proteins
PA	Phosphatidic acid
PC	phosphatidylcholine
PE	phosphatidylethanolamine
PH	pleckstrin homology
PI	phosphoinositide
PITP	phosphatidylinositol transfer protein
PKC	protein kinase C
PLA2	phospholipase A2
PM	plasma membrane
PRELI	protein of relevant evolutionary and lymphoid interest
PtdIn	phosphatidyl inositol
SHP	small heterodimer partner
SM	sphingomyelin
START	steroidogenic acute regulatory protein-related lipid transfer
TriTrypDB	database for kinetoplastid parasites
